# Geospatial analysis of *Plasmodium falciparum* serological indicators: school versus community sampling in a low-transmission malaria setting

**DOI:** 10.1186/s12916-023-03145-6

**Published:** 2024-01-23

**Authors:** Alicia Jaramillo-Underwood, Camelia Herman, Samuel E. Jean, Doug Nace, E. Scott Elder, Keri Robinson, Alaine Knipes, Caitlin M. Worrell, LeAnne M. Fox, Luccene Desir, Carl Fayette, Alain Javel, Franck Monestime, Kimberly E. Mace, Venkatachalam Udhayakumar, Kimberly Y. Won, Michelle A. Chang, Jean F. Lemoine, Eric Rogier

**Affiliations:** 1 US Centers for Disease Control and Prevention, Atlanta, GA 30329 USA; 2 Oak Ridge Institute for Science and Education (ORISE), Oak Ridge, TN 37830 USA; 3 CDC Foundation, Atlanta, GA 30308 USA; 4 Population Services International, Port-Au-Prince, Haiti; 5 The Carter Center, Atlanta, GA 30307 USA; 6 IMA World Health, Port-Au-Prince, Haiti; 7 RTI International, Port-Au-Prince, Haiti; 8 Ministère de La Santé Publique Et de La Population, Port Au Prince, Haiti; 9 Division of Digestive Diseases and Nutrition, University of Kentucky, Lexington, United States

**Keywords:** *Plasmodium falciparum*, IgG serology, Multiplex, Geospatial

## Abstract

**Background:**

Due to low numbers of active infections and persons presenting to health facilities for malaria treatment, case-based surveillance is inefficient for understanding the remaining disease burden in low malaria transmission settings. Serological data through the detection of IgG antibodies from previous malaria parasite exposure can fill this gap by providing a nuanced picture of where sustained transmission remains. Study enrollment at sites of gathering provides a potential approach to spatially estimate malaria exposure and could preclude the need for more intensive community-based sampling.

**Methods:**

This study compared spatial estimates of malaria exposure from cross-sectional school- and community-based sampling in Haiti. A total of 52,405 blood samples were collected from 2012 to 2017. Multiplex bead assays (MBAs) tested IgG against *P. falciparum* liver stage antigen-1 (LSA-1), apical membrane antigen 1 (AMA1), and merozoite surface protein 1 (MSP1). Predictive geospatial models of seropositivity adjusted for environmental covariates, and results were compared using correlations by coordinate points and communes across Haiti.

**Results:**

Consistent directional associations were observed between seroprevalence and environmental covariates for elevation (negative), air temperature (negative), and travel time to urban centers (positive). Spearman’s rank correlation for predicted seroprevalence at coordinate points was lowest for LSA-1 (*ρ* = 0.10, 95% CI: 0.09–0.11), but improved for AMA1 (*ρ* = 0.36, 95% CI: 0.35–0.37) and MSP1 (*ρ* = 0.48, 95% CI: 0.47–0.49).

**Conclusions:**

In settings approaching *P. falciparum* elimination, case-based prevalence data does not provide a resolution of ongoing malaria transmission in the population. Immunogenic antigen targets (e.g., AMA1, MSP1) that give higher population rates of seropositivity provide moderate correlation to gold standard community sampling designs and are a feasible approach to discern foci of residual *P. falciparum* transmission in an area.

**Supplementary Information:**

The online version contains supplementary material available at 10.1186/s12916-023-03145-6.

## Background

Malaria is a disease caused by infection with *Plasmodium* parasites, and global cases and deaths have declined substantially since 2000 [1], with 25 countries achieving zero locally acquired cases and many more approaching this significant milestone [2, 3]. For countries approaching elimination, lower overall parasite burden in the population and symptomatic disease prevalence in the population increases difficulty in estimating the remaining areas of *Plasmodium* transmission [4]. *P. falciparum* incidence is often estimated through passive case detection from patients presenting to health facilities, but also is accomplished through active case detection in community settings that can capture the infected, but non-treatment–seeking population [5, 6]. Active case detection is typically conducted through diagnostic testing of high-risk individuals with microscopy or rapid diagnostic tests (RDTs), but low-density parasite infections below diagnostic test limit of detection, or history of past infections, cannot be captured through these methods [7]. Similarly, parasite prevalence estimates lack sensitivity and statistical or spatial precision when numbers of parasite-positive samples are low [8, 9]. Thus, in low-transmission malaria settings, more sensitive tools are required to understand a population’s infection burden and where transmission foci remain.

Assays detecting antibodies elicited in response to *P. falciparum* infection are a sensitive indicator of personal malaria infection history, and multiple antigen targets have been identified with varying durability of seropositivity in humans [10–13]. Antibodies produced during sporozoite and liver stages of the parasite life cycle (such as circumsporozoite protein (CSP) and liver stage antigen-1 (LSA-1), respectively) can be used to estimate an individual’s *P. falciparum* exposure within the past few months [14, 15]. Conversely, IgG generated against blood stage antigens (including apical membrane antigen 1 (AMA1) and merozoite surface protein 1 (MSP1)) can remain for years, or even decades, post-exposure [14, 15]. Serological data are more commonly analyzed at the population level in order to garner historical estimates of malaria exposure in a setting (or a sub-population) [8, 12, 16]. Recent advancements in serological assay technology have allowed measurement of IgG against multiple antigens simultaneously in a serum sample, and the multiplex bead assay (MBA) has become a popular tool for this objective [17, 18].

Serological data have been collected from various survey designs to estimate malaria exposure, including population-representative surveys [19, 20] as well as those conducted at venues where persons gather [21, 22]. The convenience and accessibility of venue-based sampling presents an attractive methodology for enrollment of individuals for malaria estimates [23], but an important question for investigation is how estimates from venue-based sampling would compare to those from surveys designed to representatively sample the population.

Haiti is one of two Caribbean countries endemic for malaria, and recent cross-sectional surveys have found low levels of nationwide transmission in the country with approximately 1% or less of persons with active *P. falciparum* infection [24–26]. Investments into the nation’s health system after the 2010 earthquake and subsequent cholera epidemic resulted in intensified malaria elimination efforts [26], and surveys at sites of gathering have complemented population-representative surveys in efforts to estimate remaining burden [27]. This study compares MBA IgG data collected from population-representative community surveys in Haiti to those from school-based surveys conducted from 2012 to 2017. Geostatistical predictive modeling yielded estimates of school-based seroprevalence for comparison with those from community-based surveys, which were considered the gold standard for representative population sampling.

## Methods

### TRaC and TAS study designs and ethics

Tracking Results Continuously (TRaC) were community-based surveys conducted in 2012, 2015, and 2017 as part of The Global Fund round 8 malaria surveillance activities in Haiti. Surveys were implemented by Population Services International (PSI)-Haiti to characterize the burden of malaria across Haiti. Signed informed consent forms were obtained from all participants; for children under 15 years, verbal assent was given with documented parental consent. Enumeration areas throughout the country (*sections d'énumération*, SDE) were chosen on a proportional sampling by predicted malaria risk strata as determined by predictive modeling. A target of 20 households was randomly selected by field teams within each SDE, and all members of the household were offered the opportunity to participate. Blood was collected by fingerprick on Whatman 903 Protein Saver cards (GE Healthcare, Chicago, IL), dried overnight, and individually stored in plastic bags with desiccant at − 20 °C until blood elution and IgG assay. RDTs (CareStart HRP2; AccessBio, Somerset, NJ) were provided to all participants, and individuals testing positive were treated according to national guidelines. Blood samples were assigned unique identification numbers that were not traceable to the individual. The study protocols were approved by the Haitian Ministry of Health and approved as a non-research activity by the Center for Global Health Human Research Protection Office (HRPO), US Centers for Disease Control and Prevention (CDC; Center for Global Health determination #2015–04).

Lymphatic filariasis transmission assessment surveys (TASs) were conducted in Haiti from 2014 to 2017 with integration of malaria RDTs (First Response Malaria Histidine-Rich Protein II (HRP2), II3FRC30, Premier Medical Corporation, New Jersey) into surveys in the years 2016 and 2017 [27]. As described previously [25, 27], TASs were conducted in evaluation units (EUs) that had met World Health Organization (WHO) criteria to conduct lymphatic filariasis school-based TASs (where the net primary-school enrollment rate is ≥ 75%), and target sample size was determined by WHO-provided tables [28]. For enrollment into a TAS, school headmasters were contacted in advance regarding the survey’s purpose and asked to notify parents; children’s verbal assent was provided before sample collection. Approximately 60 µL of fingerprick blood was collected on filter papers (TropBio filter wheels, Cellabs, Sydney, Australia), dried to create a dried blood spot (DBS), and packaged individually with desiccant for later laboratory analysis at the Centers for Disease Control and Prevention in Atlanta, GA. DBS were kept at − 20 °C until laboratory analysis. The study protocol was approved by the National Bioethics Committee of Haiti, and this activity was considered a program evaluation activity by the CDC Human subjects office (#2014–256). Persons consented to future laboratory testing of DBS, and CDC laboratory staff did not have access to any personal identifiers.

### Plasmodium falciparum recombinant antigens

Purified recombinant antigens were covalently linked to xMAP microspheres (Luminex Corp, Austin, TX), as described previously [29]. Three antigens were utilized for all surveys: *P. falciparum* liver stage antigen 1 Pl1043 epitope peptide (LSA-1, coupled at pH 5 at 60 µg/mL) [30]; recombinant merozoite surface protein 1 19kD fragment (MSP1, coupled at pH 5 at 20 µg/mL) [31]; and recombinant apical membrane antigen 1 N-terminal region (AMA1, coupled at pH 5 at 20 µg/mL) [31]. These three *P. falciparum* antigens have been widely used for previous malaria serological studies by multiple groups, and represent the evaluation of long-term IgG responses (MSP1 19kD region, AMA1 N-terminal region) [8, 10, 14–16] as well as host IgG responses with shorter duration (LSA-1 Pl1043 epitope) [14, 15].

### Sample preparation and multiplex IgG detection assay

A single six-mm DBS punch of the Whatman 903 card for TRaC surveys or a single DBS tab of the TropBio filter wheel for TAS surveys was rehydrated in blocking buffer: PBS pH 7.2; 0.5% polyvinyl alcohol (Sigma-Aldrich, St. Louis, MO); 0.5% polyvinylpyrrolidine (Sigma-Aldrich); 0.1% casein (ThermoFisher, Waltham, MA); 0.5% BSA (Sigma-Aldrich); 0.3% Tween-20; 0.05% sodium azide; and 3 µg/mL *E. coli* extract to prevent non-specific binding. For the multiplex IgG detection assay, samples were diluted to a final concentration of 1:200 whole blood, which is approximately a 1:400 serum dilution with the assumption of 50% hematocrit in whole blood.

IgG detection through MBA was performed as described previously [32]. Briefly, samples were incubated with a mixture of all three bead regions in reagent buffer (PBS, 0.05% Tween-20, 0.5% BSA, 0.02% NaN_3_) for 90 min at room temperature under gentle shaking protected from light in MultiScreen-BV filter plates (MilliporeSigma, Burlington, MA) or Bio-Plex Pro plates (Bio-Rad, Hercules, CA). After three washes (wash buffer: PBS, 0.05% Tween-20), beads were incubated with 50 µL biotinylated detection antibody (a mixture of 1:500 anti-hIgG and 1:625 anti-hIgG4, both produced by Southern Biotech, Birmingham, AL) for 45 min with the same incubation conditions as above. After three washes, 50 µL of a 1:250 × dilution of streptavidin–phycoerythrin (Invitrogen, Waltham, MA) was added to all wells for a 30-min incubation. After three washes, sample beads were incubated with 50 µL reagent buffer for 30 min to remove loosely-bound IgG, washed once, and resuspended in 100 µL PBS. Assay plates were briefly shaken and read on a Bio-Plex 200 machine (Bio-Rad) at a high RP1 target or on a MAGPIX machine (Luminex Corp) by generating the median fluorescence intensity (MFI) for 50 beads. The final measure, denoted as MFI-bg, was reported by subtracting MFI values from beads on each plate only exposed to sample diluent during the sample incubation step. Malaria IgG positive and negative samples were included in duplicate on each assay plate to ensure appropriate plate reading. The MFI-bg threshold for true positive IgG assay signal for each survey was ascertained if the sample MFI-bg was higher than the mean + 3 SD of the MFI-bg signal of a panel of known IgG negative DBS samples.

### Statistical analysis

Data analysis was performed in SAS (version 9.4; SAS Institute Inc., Cary, USA). For comparison purposes, covariates of interest included in the analysis were only those that had been collected for both TAS and TRaC studies. For the TRaC study, age was classified into approximately evenly distributed groups. For TASs, only children ages 6 and 7 were included. To assess the assumption that TRaC seroprevalence was consistent across the 2012–2017 study period, a reversible catalytic model was fit to seropositivity by age data for AMA1 and MSP1 using 15 age categories. Estimates for serological conversion rate (SCR) and serological reversion rate (SRR) for each survey year were directly derived from the likelihood model [16].

Geostatistical analysis of seroprevalence to each antigen was conducted using the R-INLA package [33] in R Statistical Software (version 4.2.0; R Foundation for Statistical Computing, Vienna, Austria). Covariates have known or putative effects on malaria exposure (Additional file 1: Tab S1). Data values for each covariate were sampled at each TAS/TRaC survey location’s GPS coordinates using QGIS (version 3.20.3-Odense). For temporal covariates, annual averages from the latest year of data available were used. All covariates were standardized and centered to facilitate model convergence, and none were omitted from the models to allow for direct comparison between studies and to evaluate all effects on malaria exposure.

Geostatistical models of seroprevalence to LSA-1, AMA1, and MSP1 for each study.

were fit using a Bayesian framework, whereby *P(x*_*i*_*)* was seroprevalence at survey locations *x*_*i*_*, i* = *1, …, n*, and the number of seropositive participants, *Y*_*i*_, out of the number of *N*_*i*_ participants surveyed at a location was assumed to follow a binomial distribution:

$${Y}_{i}|P\left({x}_{i}\right)\sim Binomial\left({N}_{i}, P\left({x}_{i}\right)\right)$$

The seroprevalence at each location was then linked to its linear predictors through the logit:

$$logit\left(P\left({x}_{i}\right)={\beta }_{0}+d{\left({x}_{i}\right)}^{\prime}\beta +{S(x}_{i}\right),$$

where, for each model, *β*_*0*_ was the intercept, $d{\left({x}_{i}\right)}^{\prime}\beta$ represented the vector of location-specific covariate effects, and ${S(x}_{i})$ was the spatial random effect, modeled using a Matérn covariance function with a stochastic partial difference equation (SPDE) (Additional file 2: Fig. S1) in integrated nested Laplace approximation (INLA). Models were validated splitting datasets into 75% training values and 25% test values. Results were extracted as mean estimated beta coefficients and as continuous surfaces of seroprevalence with a pixel size of 0.9 km^2^. The latter were imported into QGIS for sampling of predicted values for all coordinates in Haiti (*n* = 32,735) and estimating mean seroprevalence within communes (*n* = 134). Spearman’s rank correlation coefficients compared predictions at points and communes.

## Results

**Fig. 1:**
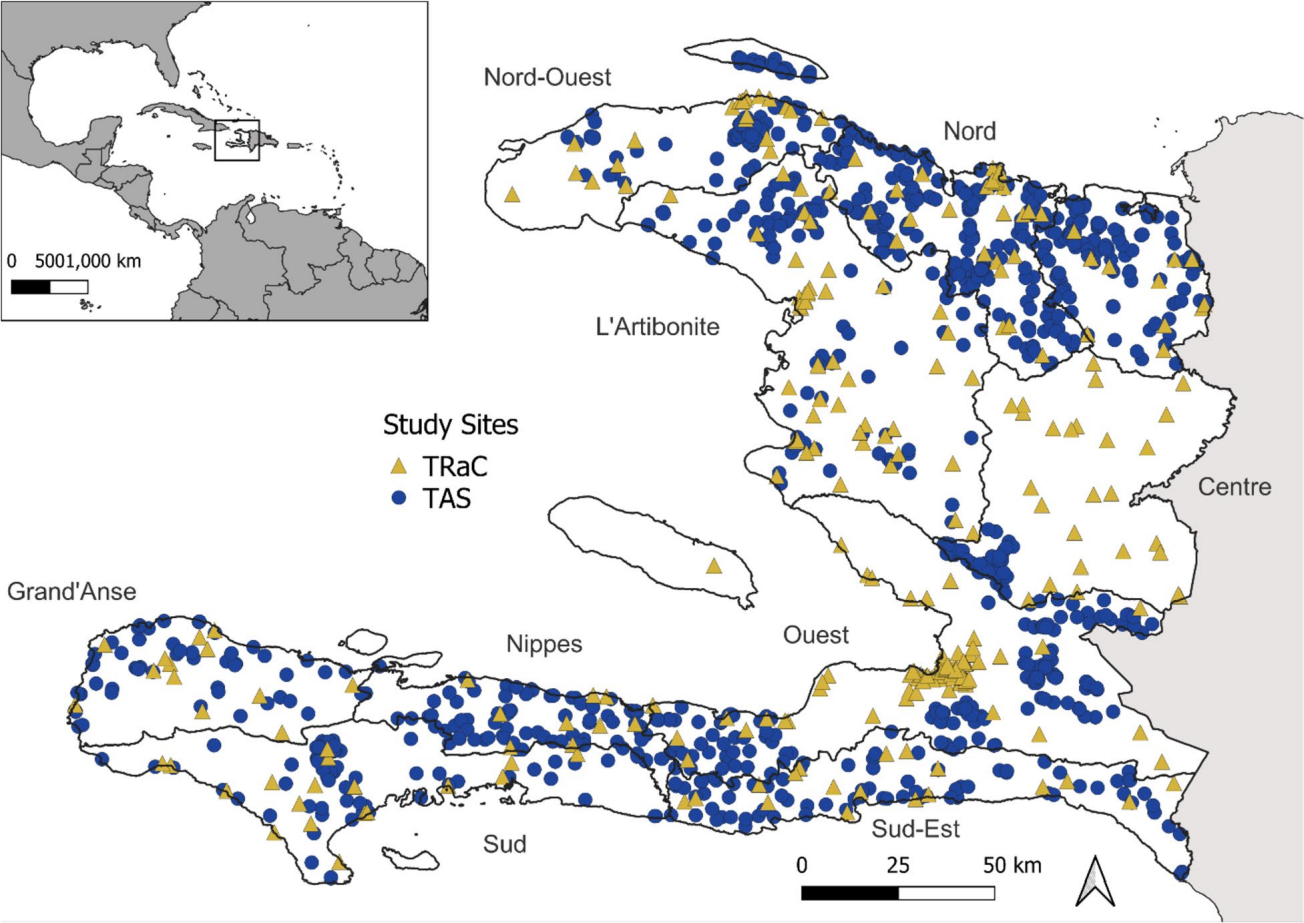
Study sites across Haiti. Tracking Results Continuously (TRaC; *n* = 324) sites are represented by yellow triangles and transmission assessment survey (TAS; *n* = 1163) sites by blue dots. Names of Haitian departments are provided; outlines represent department boundaries

From the 2014–2017 Haiti TASs, 36,094 children ages 6–7 years from 1163 schools provided DBS for IgG detection (Fig. 1, Table 1). Through Haiti TRaC surveys in 2012, 2015, and 2017, 16,311 participants of all ages enrolled from 324 communities (SDEs) (Fig. 1, Table 1). Of 16,063 children with *P. falciparum* RDT results from TASs, 18 (0.1%, 95% CI: 0.0–0.1%) tested positive; of 15,754 persons from TRaC with RDT results, 99 (0.6%, 0.5–0.8%) tested positive. Due to these low numbers, no clear spatial pattern was observed in RDT positives from both surveys (Additional file 2: Fig. S2). For the years when tests were performed, RDT prevalence was low (≤ 1% positivity) across time for both survey types (Additional file 1: Tab S2).

**Table 1:** Raw data summary of *P. falciparum* serology samples collected from two study populations in Haiti

	**Total (*N*)**		**LSA-1** **seropositive**		**AMA1** **seropositive**		**MSP1** **seropositive**	
	**TRaC**	**TAS**	**TRaC**	**TAS**	**TRaC**	**TAS**	**TRaC**	**TAS**
			*n* (%)	*n* (%)	*n* (%)	*n* (%)	*n* (%)	*n* (%)
**Participants**	16,311	36,094	240 (1.5%)	137 (0.4%)	2449 (15.0%)	1386 (3.8%)	2899 (17.8%)	1635 (4.5%)
**Survey locations**	324	1163						
**Age (years)**^a^								
< 6	3381	–	17 (0.5%)	–	122 (3.6%)	–	144 (4.3%)	–
6 to 15	3724	36,094	10 (0.3%)	137 (0.4%)	290 (7.8%)	1386 (3.8%)	391 (10.5%)	1635 (4.5%)
16 to 30	4437	–	48 (1.1%)	–	712 (16.1%)	–	931 (21.0%)	–
> 30	4492	–	161 (3.6%)	–	1,286 (28.6%)	–	1,383 (30.8%)	–
**Sex**								
Male	5974	18,019	101 (1.7%)	69 (0.4%)	874 (14.6%)	734 (4.1%)	1000 (16.7%)	865 (52.9%)
Female	9757	18,074	133 (1.4%)	68 (0.4%)	1491 (15.3%)	652 (3.6%)	1790 (18.4%)	770 (47.1%)
**RDT**								
Positive	99	18	11 (11.1%)	4 (22.2%)	41 (41.4%)	17 (94.4%)	42 (42.4%)	17 (94.4%)
Negative	15,655	16,045	226 (1.4%)	24 (0.1%)	2354 (15.0%)	400 (2.5%)	2781 (17.8%)	437 (2.7%)
**Department**								
L’Artibonite	2886	3153	50 (1.7%)	3 (0.1%)	460 (15.9%)	83 (2.6%)	566 (19.6%)	81 (2.6%)
Centre	1381	1225	44 (3.2%)	28 (2.3%)	387 (28.0%)	283 (23.1%)	371 (26.9%)	338 (27.6%)
Grand’Anse	674	1608	22 (3.3%)	30 (1.9%)	128 (19.0%)	169 (10.5%)	122 (18.1%)	250 (15.6%)
Nippes	583	2454	11 (1.9%)	4 (0.2%)	94 (16.1%)	141 (5.8%)	127 (21.8%)	163 (6.6%)
Nord	1481	9702	13 (0.9%)	23 (0.2%)	207 (14.0%)	210 (2.2%)	259 (17.5%)	215 (2.2%)
Nord-Est	530	5319	3 (0.6%)	21 (0.4%)	65 (12.3%)	192 (3.6%)	77 (14.5%)	282 (5.3%)
Nord-Ouest	1024	2806	9 (0.9%)	5 (0.2%)	116 (11.3%)	92 (3.3%)	123 (12.0%)	91 (3.2%)
Ouest	5714	5459	67 (1.2%)	4 (0.1%)	688 (12.0%)	62 (1.1%)	867 (15.2%)	37 (0.7%)
Sud	1069	2188	19 (1.8%)	10 (0.5%)	212 (19.8%)	84 (3.8%)	267 (25.0%)	123 (5.6%)
Sud-Est	852	2180	0 (0.0%)	9 (0.4%)	78 (9.2%)	70 (3.2%)	102 (12.0%)	55 (2.5%)

*TRaC* Tracking Results Continuously, *TAS* Transmission assessment survey

^a^TAS participants were between the ages of 6 and 7 years only

**Fig. 2:**
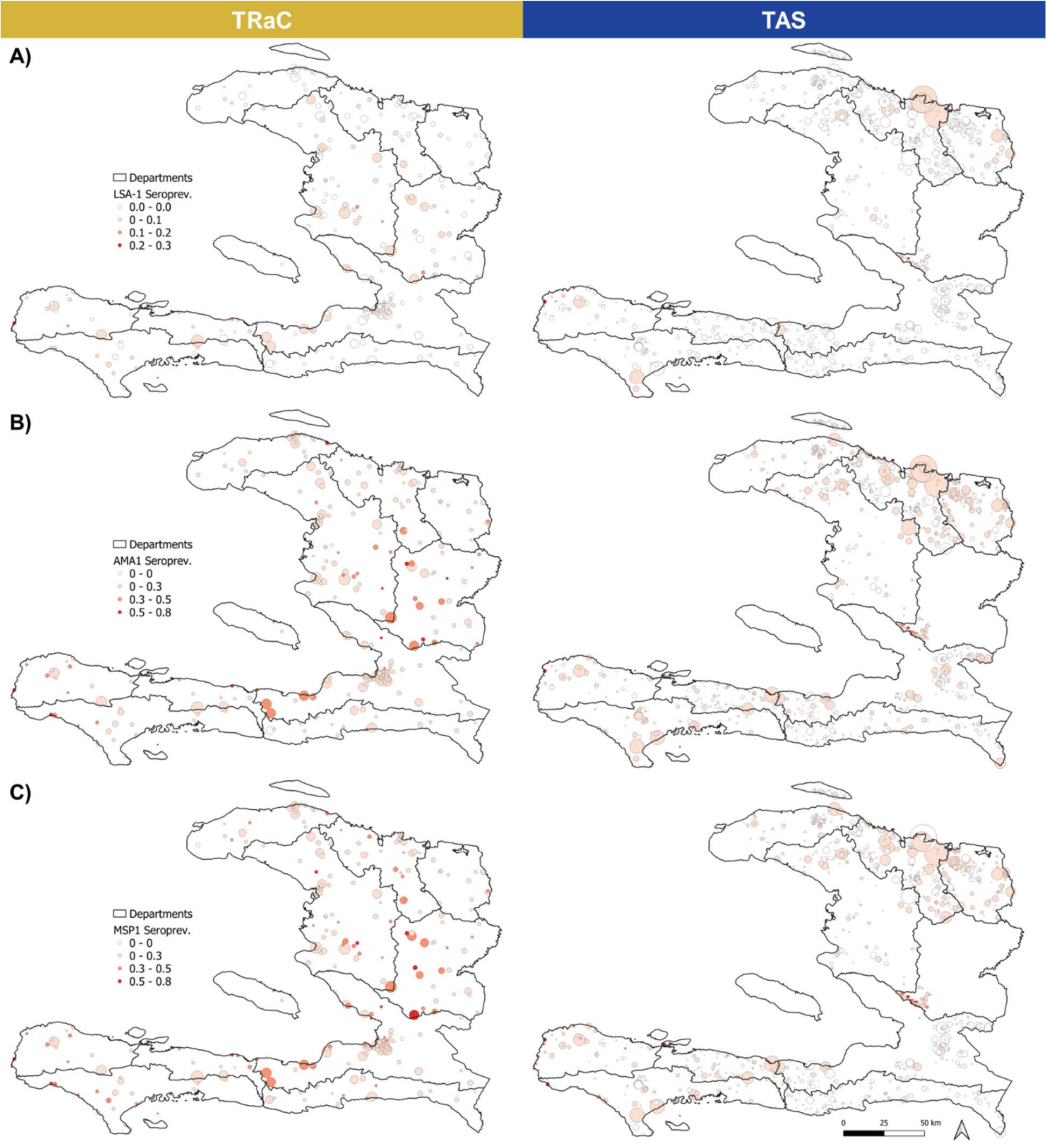
Observed seroprevalence by study site. Maps are grouped based on seroprevalence to **A** liver stage antigen-1 (LSA-1), **B** apical membrane antigen 1 (AMA1), and **C** merozoite surface protein 1 (MSP1). Tracking Results Continuously (TRaC) sites are on the left-hand side and transmission assessment survey (TAS) on the right. Circle sizes are proportional to the number of participants surveyed at each site. Darker red shading indicates higher seroprevalence; white fill indicates zero participants were seropositive at a given site. Department boundaries are outlined

IgG seropositivity to the liver-stage antigen LSA-1 was the lowest among all antigens included in the multiplex assay at 1.5% (95% CI: 1.3–1.7%) for TRaC and 0.4% (0.3–0.4%) for TAS. Seropositivity to AMA1 was 15.0% (14.5–15.6%) for TRaC, and 3.8% (3.6–4.0%) for TAS; similarly, seropositivity to MSP1 was 17.8% (17.2–18.4%) for TRaC and 4.5% (4.3–4.7%) for TAS. Seroprevalence curves and seropositivity estimates were consistent among TRaC survey years (Additional file 2: Fig. S3, Additional file 1: Tab S3). Most TAS schools (91%) and TRaC SDEs (60%) had no participants seropositive to LSA-1, whereas at least 44% of schools and SDEs had persons seropositive to AMA1 or MSP1 (Fig. 2). TRaC sites had higher median population density, sparser vegetation, and lower travel time to urban centers (Additional files 1: Tab S4 and S5). Elevation, temperature, rainfall, and distance to the nearest river, lake, or stream were generally comparable between studies. Individual-level correlations in the IgG level among LSA-1, MSP1, and AMA1 antigens are shown in Additional file 2: Fig. S4 and showed no marked correlation among IgG levels to these three *P. falciparum* targets.

**Fig. 3:**
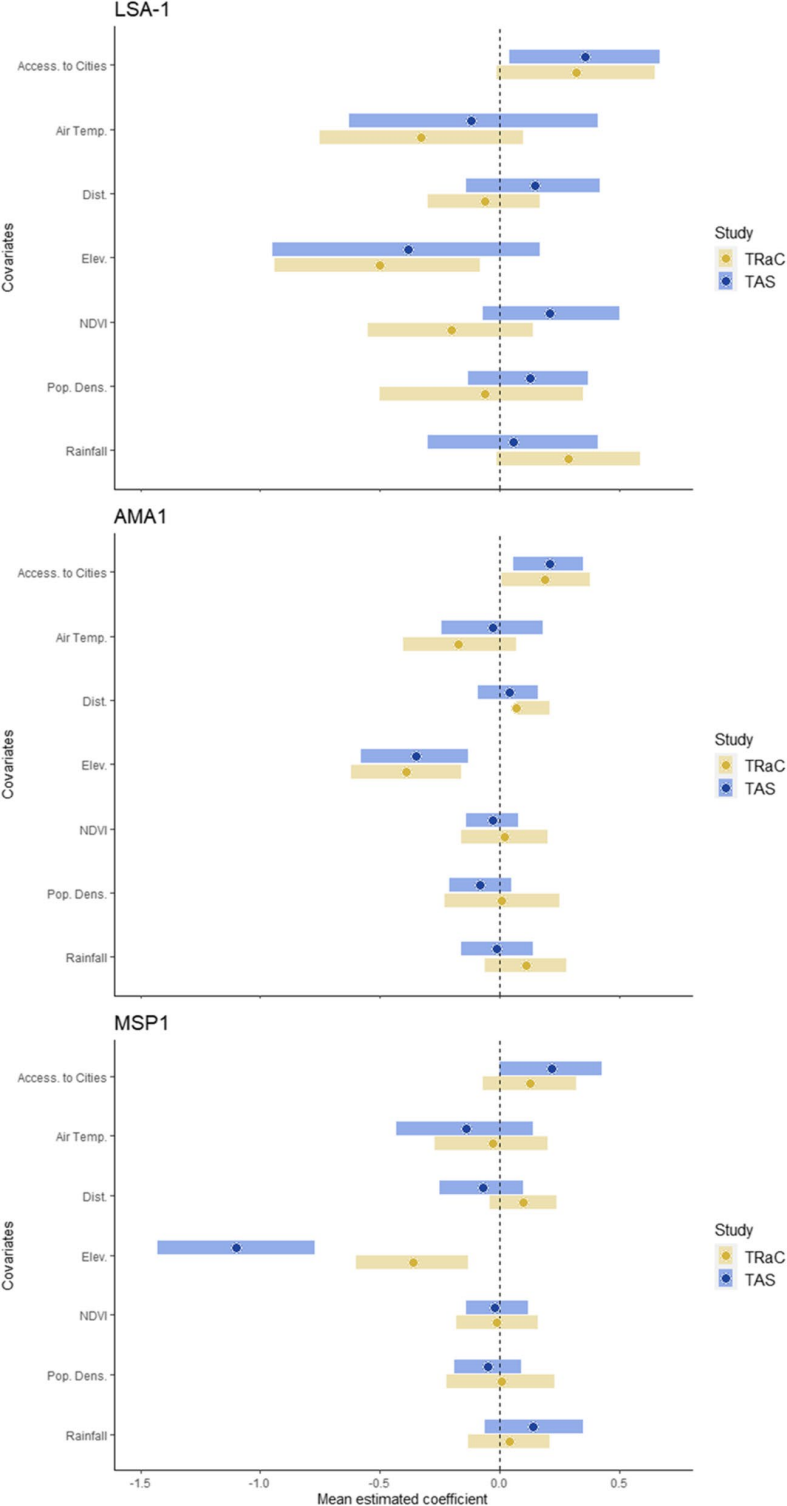
Mean estimated coefficients of covariates with 95% credible intervals for seroprevalence to liver stage antigen-1 (LSA-1), apical membrane antigen 1 (AMA1), and merozoite surface protein 1 (MSP1). All covariates have been centered and standardized. Accessibility to cities is represented by mean travel time to urban centers, such that an increase in this variable indicates a more rural location. Dots represent mean estimated beta coefficients for covariates used in integrated nested Laplace approximation (INLA) models with the stochastic partial difference equation (SPDE) approach; bars are the coefficients’ 95% credible intervals. A vertical line at 0.0 represents a null effect

 From spatial predictive modeling, many covariates had similar relationships with seroprevalence outcomes between the two survey types (Fig. 3, Additional file 1: Tab S6). Increased air temperature was consistently associated with decreased seroprevalence to *P. falciparum* targets, while greater travel time to urban centers (i.e., a more rural setting) was positively associated with seroprevalence. There was a negative association between elevation and seroprevalence across both survey types. Increases in rainfall and distance to the nearest body of water were generally associated with increases in seroprevalence. Based on cross-validation results, the R-INLA model of seroprevalence to LSA-1 in the TAS venue-based survey had the lowest prediction ability (Additional file 2: Fig. S5).

**Fig. 4:**
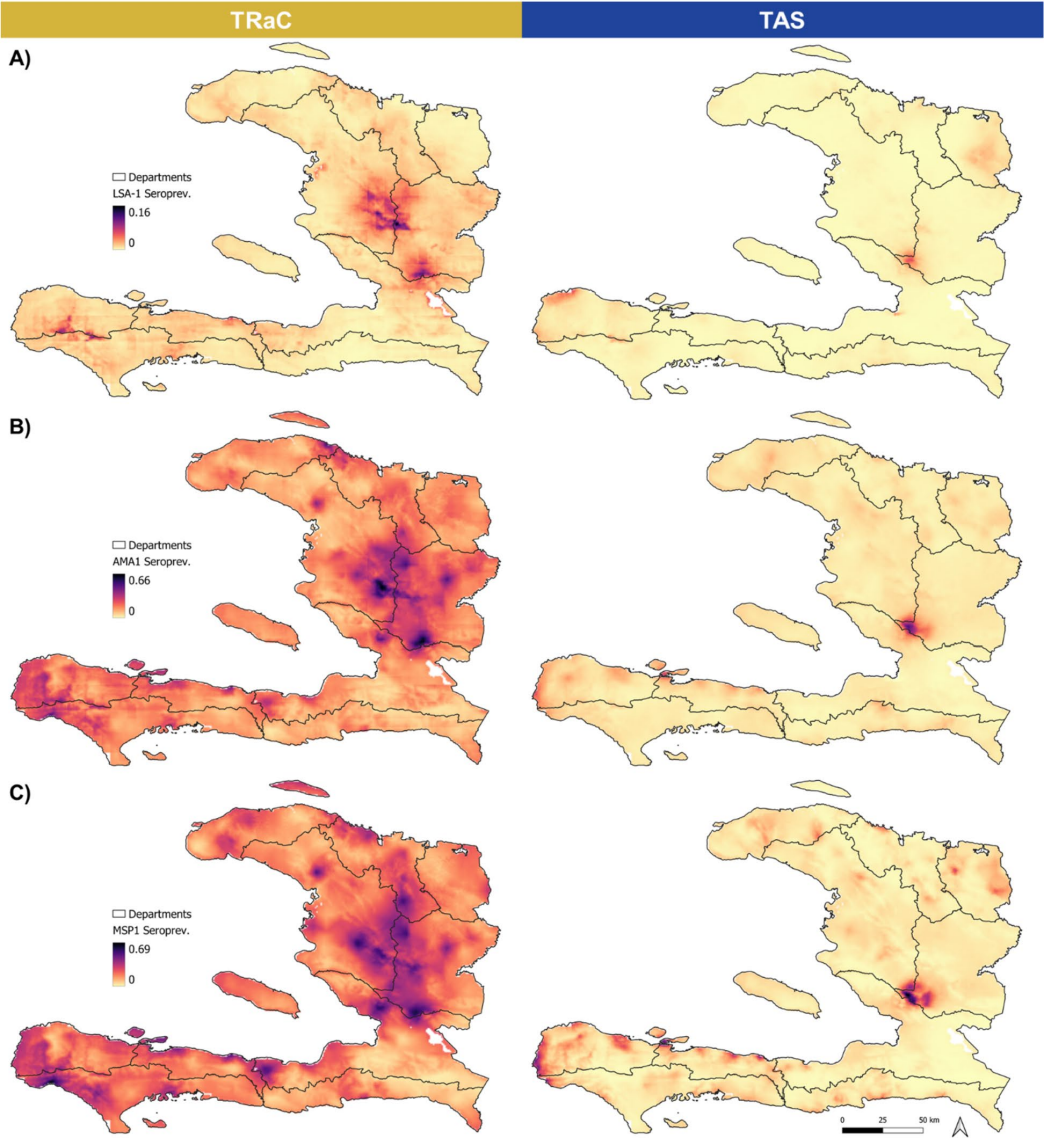
Predicted seroprevalence across Haiti to **A** liver stage antigen-1 (LSA-1), **B** apical membrane antigen 1 (AMA1), and **C** merozoite surface protein 1 (MSP1). Tracking Results Continuously (TRaC) predictions are on the left panels and transmission assessment survey (TAS) on the right. Darker red to purple shading indicates higher seroprevalence, while paler shades of red to yellow indicate lower seroprevalence. Scale of shading is consistent for the same antigen target between two survey types. Department boundaries are outlined, and scale bar and north arrow illustrated. Pixel size is approximately 0.9 km^2^. Predicted seroprevalence was modeled by linking the observed seroprevalence at TAS or TRaC survey locations to environmental covariate data spanning all coordinate points across Haiti. Predicted seroprevalence was extracted as a continuous surface for mapping

Due to the enrollment of older ages in TRaC, observed and predicted seroprevalence was higher and more geographically widespread for TRaC (Fig. 4). Correlations were weakest for LSA-1 (*ρ*_points_ = 0.10, 95% CI: 0.09–0.11; *ρ*_communes_ = -0.03, 95% CI: − 0.20–0.14) and strongest for MSP1 (*ρ*_points_ = 0.48, 0.47–0.49; *ρ*_communes_ = 0.49, 0.34–0.61) when examining strength of correlations between predicted seroprevalence values (Additional file 2: Fig S6). The MSP1 target had the highest observed and predicted seroprevalence across both studies. The AMA1 target, with lower values relative to MSP1, had lower correlations (*ρ*_points_ = 0.36, 0.35–0.37; *ρ*_communes_ = 0.40, 0.25–0.53) (Table 2). While higher population seropositivity was observed if persons were considered “seropositive” when IgG positive to either AMA1 or MSP1 targets (23.5% for TRaC and 6.8% for TAS), there was no improvement in correlations of predicted estimates (*ρ*_points_ = 0.43, 0.42–0.43; *ρ*_communes_ = 0.46, 0.32–0.58).

**Table 2:** Spearman’s rank correlation coefficient, *ρ*, and 95% confidence intervals for TAS versus TRaC surveys

Antigen	Seroprevalence by coordinate points across Haiti *ρ* (95% CI)	Seroprevalence by aggregated commune estimates *ρ* (95% CI)
LSA-1	0.10 (0.09–0.11)	− 0.03 (− 0.20–0.14)
AMA1	0.36 (0.35–0.37)	0.40 (0.25–0.53)
MSP1	0.48 (0.47–0.49)	0.49 (0.34–0.61)
AMA1 and/or MSP1	0.43 (0.42–0.43)	0.46 (0.32–0.58)

*TRaC* Tracking results continuously, *TAS* Transmission assessment survey

## Discussion

This study utilized geospatial modeling to predict seroprevalence to different *P. falciparum* antigens to compare two sampling methodologies: population-representative household-based enrollment (TRaC surveys), and school-based enrollment (TASs). Regions approaching *P. falciparum* elimination are faced with epidemiological challenges to accurately identify the remaining foci of transmission [2, 4, 7]. In low-transmission settings where persons presenting to healthcare settings with malaria infection is rare, seropositivity to *P. falciparum* antigens extends the window of a “positive” malaria exposure and can indicate where (and within whom) infections are still occurring [8, 34, 35]. As malaria transmission decreases in a population, resources become diverted away from malaria programs in favor of matters considered more important to public health. For this and other reasons, generating accurate serological estimates from venue-based sampling or survey designs implemented by non-malaria groups could increase efficiency and augment malaria epidemiology in these settings.

Among the years of sample collection, tests for active *P. falciparum* infection by RDT confirmed a low prevalence of *P. falciparum* in Haiti, and each cross-sectional survey found RDT positivity of 1.0% or less. The same paucity of infections through cross-sectional surveys with RDTs has been observed in other low-transmission settings [37, 38]. These studies have shown reductions in RDT diagnostic performance among non-treatment–seeking persons [36, 37], and demonstrated that RDTs cannot reliably detect asymptomatic infections accounting for a significant proportion of *P. falciparum* burden in these populations [38, 39]. These RDT results diverge with serological findings from this study, which identified persons with anti-*P. falciparum* IgG throughout Haiti and provided augmented numbers of positives to allow for statistical modeling. Much like RDT positivity, seropositivity to LSA-1, reflecting *P. falciparum* exposure in the past few months [15], was low across both survey types, reaching a maximum seropositivity of 1.5% for persons enrolled in TRaC. Cumulative exposure to malaria over time increases the proportion of persons seropositive to *P. falciparum* antigens in a population, and this is especially profound for IgG targets such as AMA1 and MSP1 that are known to provide “long-lived” B cell responses and seropositivity for years after an infection [8, 16, 40]. This was noted by the AMA1 and MSP1 seroprevalence curves for the TRaC surveys, which showed no change in exposure estimates from 2012 to 2017. Because TASs only included children 6 and 7 years of age, seroprevalence to all *P. falciparum* antigens was overall lower when compared to persons enrolled in TRaC surveys, which were inclusive of all ages.

Results from INLA-SPDE models showed that certain environmental variables yielded consistent relationships between survey types. Elevation had a negative association with all serological outcomes. Multiple studies have also observed this association with increased elevation and decreased parasite prevalence [34], malaria incidence [41], and seroprevalence to *P. falciparum* antigens [16, 19], as lower elevations allow for more suitable temperature and moisture conditions for the *Anopheles* vector to breed [42]. Increased travel time to urban centers also had a consistently positive relationship with seropositivity. Greater mean travel time to urban centers would indicate more rural survey locations, and rural residence has been consistently associated with greater *Anopheline* habitat and malaria transmission when compared to urban settings, as well as generally lower socioeconomic indicators for rural residents [43, 44]. In line with improved conditions for mosquito habitat, rainfall had a positive relationship with *P. falciparum* seropositivity for all models except for one. This positive relationship has also been noted in multiple malaria studies [45, 46]. Air temperature was found to have a consistently negative association with seropositivity. Previous studies have found positive and negative associations between air temperature and malaria incidence; given that temperature interacts with other factors influencing malaria prevalence, these conflicting findings are not unexpected [47, 48].

Spatial patterns predicted from this analysis are reflected in maps produced by Cameron et al., which show higher clinical incidence in central and southwestern Haiti compared to other areas [49]. A higher risk of transmission has also previously been reported in the southern coastal regions of Haiti [50]. Between the TRaC and TAS surveys, the AMA1 and MSP1 IgG targets had both the highest observed seropositivity and highest correlations when comparing predicted seropositivity. However, if defining “seropositivity” as IgG positive to either long-term IgG target (in the same manner as previous studies [21, 35]), while the proportion of those who were seropositive increased, a correlation between predictions was not improved. This may reflect a tradeoff between sensitivity and specificity of a true “seropositive” designation, whereas a higher number of positives may be compounded by more false positives through a multi-antigen approach. While the magnitude of the correlation between TRaC and TAS predicted seroprevalence was similar whether comparing points or communes, correlation coefficients for coordinate points had narrower confidence intervals than those for communes. This emphasizes the utility of high-resolution data points in geostatistical analysis over data aggregated to administrative boundaries. Moderate agreement in predictive spatial outputs between the two survey types shows some utility in using venue-based sampling methods as a proxy for sampling designed to be representative of an entire population.

Limitations to this study include limited sociological and individual covariates that were common between TRaC and TAS surveys that could be used for predictive modeling. While TRaC surveys were designed to be nationally representative with an exclusive focus on malaria, TAS surveys were powered based on estimated lymphatic filariasis antigenemia within enumeration units throughout Haiti. More comprehensive TAS sampling with consideration for malaria may have improved correlations between predictive outputs, in addition to geographical representation of the same study areas and inclusion of a wider age range for more extensive serological data analysis. A wider age range would also be ideal when using venue-based sampling methods as a proxy for population-level transmission or exposure estimates. This study used a limited panel of *P. falciparum* IgG targets, and dozens of potential targets are now available [41]. An expanded panel may find other antigen targets (or combinations thereof) with better correlations among survey types.

In settings of low *P. falciparum* transmission, serological data can augment epidemiological information beyond what tests for active malaria infection can provide. While various methodologies can use serological data to produce malaria exposure estimates, sampling at places of congregation could provide a pragmatic approach as countries seek elimination strategies.

## Conclusions

Along with carefully considered epidemiological designs, potential assays that include more immunogenic antigen targets, which yield higher population rates of seropositivity, are a feasible approach to discern the foci of residual *P. falciparum* transmission in an area. Venue-based sampling methods to generate serological data may also have utility for non-*Plasmodium* infectious diseases, and this strategy should be tested further in other global settings.

### Supplementary Information

**Additional file 1: Table S1.** Remote sensing data: resolutions, units, and sources. **Table S2.** Prevalence of positive rapid diagnostic test results with 95% confidence intervals by survey year for Tracking Results Continuously versus Transmission Assessment Surveys. **Table S3.** Observed seropositivity to P. falciparum antigens by survey year with 95% confidence intervals for community members surveyed for Tracking Results Continuously, 2012-2017. **Table S4.** Descriptive statistics of survey-site temporal covariates. **Table S5.** Descriptive statistics of survey-site static covariates. **Table S6.** Mean estimated coefficients of covariates with 95% credible intervals.

**Additional file 2: Fig S1.** Triangulated meshes used to build the stochastic partial difference equation (SPDE) models. **Figure S2.** Number of participants with a positive rapid diagnostic test (RDT) for malaria by study site. **Figure S3.** IgG seropositivity by age for Tracking Results Continuously (TRaC) community surveys in Haiti, 2012-2017. **Figure S4.** Correlation in the observed IgG levels among the LSA-1, MSP1, and AMA1 antigens utilized in this study for both survey types. **Figure S5.** Correlations between observed and predicted seroprevalence to (A) LSA-1, (B) AMA1, and (C) MSP1 based on model validation. **Figure S6.** Predicted seroprevalence across Haiti to (A) LSA-1, (B) AMA1, and (C) MSP1, aggregated for each commune.

## Data Availability

The datasets used and/or analyzed during the current study are available from the corresponding author on reasonable request.

## Abbreviations

AMA1: Apical membrane antigen 1
CDC: Centers for disease control and prevention
CSP: Circumsporozoite protein
DBS: Dried blood spot
IgG: Immunoglobulin G
INLA: Integrated nested Laplace approximation
LSA-1: Liver-stage antigen-1
MBA: Multiplex bead assay
MFI: Median fluorescence intensity
MFI-bg: Median fluorescence intensity minus background
MSP1: Merozoite surface protein 1
PSI: Population Services International
RDT: Rapid diagnostic test
SCR: Serological conversion rate
SDE: *Sections* *d'énumération*
SPDE: Stochastic partial difference equation
SRR: Serological reversion rate
TAS: Transmission assessment survey
TRaC: Tracking Results Continuously
WHO: World Health Organization
