# Supplementary material for: Geospatial analysis of Plasmodium falciparum serological indicators: school versus community sampling in a low-transmission malaria setting
Source: BMC Med. 2024 Jan 23;22:31. doi: 10.1186/s12916-023-03145-6 (PMC10804471; doi:10.1186/s12916-023-03145-6)
Supplement: Supplementary file 1 — Additional file 1: Table S1. Remote sensing data: resolutions, units, and sources. Table S2. Prevalence of positive rapid diagnostic test results with 95% confidence intervals by survey year for Tracking Results Continuously versus Transmission Assessment Surveys. Table S3. Observed seropositivity to P. falciparum antigens by survey year with 95% confidence intervals for community members surveyed for Tracking Results Continuously, 2012-2017. Table S4. Descriptive statistics of survey-site temporal covariates. Table S5. Descriptive statistics of survey-site static covariates. Table S6. Mean estimated coefficients of covariates with 95% credible intervals. [file 12916_2023_3145_MOESM1_ESM.docx]

**Additional File 1**

**Table S1. Remote sensing data: resolutions, units, and sources**

| **Covariate** | **Unit** | **Spatial Resolution** | **Temporal Resolution** | **Source** |
| --- | --- | --- | --- | --- |
| Accessibility to cities | mean travel time in minutes to urban center | 1 km^2^ | N/A | Weiss et al., 2018^1^ |
| Air temperature | °C | 0.05 decimal degrees | 1 month | Accessible by figshare^2^ |
| Distance to water* | m | N/A | N/A | Digital Chart of the World^3^ |
| Elevation | m | 90 m | N/A | CGIAR SRTM^4^ |
| Normalized difference vegetation index (NDVI) | ratio | 250 m | 1 dekad | USGS^5^ |
| Population density | population / km^2^ | 1 km^2^ | 1 year | WorldPop^6^ |
| Rainfall | mm | 0.05 x 0.05 degree | 1 dekad | CHIRPS^7^ |

* Nearest mapped river, lake, or stream

^1^ Weiss, D. J., et al. (2018). "A global map of travel time to cities to assess inequalities in accessibility in 2015." Nature 553(7688): 333-336

^2^https://doi.org/10.6084/m9.figshare.c.4081802.v1

^3^https://docs.generic-mapping-tools.org/6.1/datasets/dcw.html

^4^https://bigdata.cgiar.org/srtm-90m-digital-elevation-database/

^5^https://earlywarning.usgs.gov/fews/product/447

^6^https://www.worldpop.org/geodata/listing?id=77

^7^https://data.chc.ucsb.edu/products/CHIRPS-2.0/global_monthly/tifs/

**Table S2. Prevalence of positive rapid diagnostic test results with 95% confidence intervals by survey year for Tracking Results Continuously versus Transmission Assessment Surveys**

| **Survey Year** | **TRaC % RDT Positive (Percent, 95% CI)** | **TAS RDT Positive**  **(Percent, 95% CI)** |
| --- | --- | --- |
| 2012 | 1.0 (0.7–1.3) | ***** |
| 2014 | ***** | ****** |
| 2015 | 0.6 (0.3–0.8) | ****** |
| 2016 | ***** | 0.2 (0.1–0.2) |
| 2017 | 0.4 (0.2–0.5) | 0.0 (0.0–0.0) |

*****Survey year not applicable

**RDTs were not deployed for TAS surveys in these years

**Table S3. Observed seropositivity to *P. falciparum* antigens by survey year with 95% confidence intervals for community members surveyed for Tracking Results Continuously, 2012-2017.**

| **Survey Year** | **% LSA-1 Seropositive**  **(95% CI)** | **% AMA1 Seropositive**  **(95% CI)** | **% MSP1 Seropositive**  **(95% CI)** |
| --- | --- | --- | --- |
| 2012 | 1.0 (0.8–1.3) | 13.5 (12.6–14.4) | 17.5 (16.5–18.5) |
| 2015 | 1.9 (1.5–2.3) | 18.8 (17.7–19.9) | 22.1 (20.9–23.3) |
| 2017 | 1.5 (1.2–1.8) | 13.7 (12.8–14.5) | 14.9 (14.1–15.8) |

**Table S4. Descriptive statistics of survey-site temporal covariates**

| **Covariate** | **TRaC** | | | **TAS** | | | |
| --- | --- | --- | --- | --- | --- | --- | --- |
|  | **2012** | **2015** | **2017** | **2014** | **2015** | **2016** | **2017** |
| Air temperature (◦C) | 26.1 (25.1–26.3) | 26.5 (25.4–26.3) | 26.4 (25.4–26.7)* | 25.5 (24.6–26.3) | 25.6 (24.8–26.5) | 25.7 (24.8–26.5) | 25.6 (24.8–26.4)* |
| NDVI (ratio) | 0.66 (0.46–0.75) | 0.64 (0.45–0.73) | 0.67 (0.46–0.76) | 0.72 (0.64–0.77) | 0.71 (0.63–0.76) | 0.74 (0.67–0.78) | 0.75 (0.67–0.79) |
| Population density (people/km^2^) | 728 (252–11,750) | 791 (237–11,902) | 799 (234–11,849) | 373 (200–750) | 391 (195–796) | 391 (200–797) | 379 (197–800) |
| Rainfall (mm) | 112 (87–133) | 81 (65–95) | 134 (103–156) | 84 (64–101) | 78 (64–99) | 116 (90–142) | 129 (101–152) |

Data are median (IQR) of annual average values for all study sites.

*Data from 2017 were extrapolated using all previously available data (2003-2016). Air temperature data used in analysis were from 2016.

NDVI: Normalized difference vegetation index

**Table S5. Descriptive statistics of survey-site static covariates**

| **Covariate** | **TRaC** | **TAS** |
| --- | --- | --- |
| Elevation (m) | 106 (32–328) | 188 (45–437) |
| Accessibility to cities (mean travel time in minutes to urban center) | 13 (0–47) | 30 (17–52) |
| Distance to water* (km) | 2.4 (0.9–4.2) | 1.7 (0.7–3.5) |

Data are median (IQR) of values for all study sites.

*Nearest mapped river, lake, or stream

**Table S6. Mean estimated coefficients of covariates with 95% credible intervals**

| **Covariate** | **LSA-1** | | **AMA1** | | **MSP1** | |
| --- | --- | --- | --- | --- | --- | --- |
|  | **TRaC** | **TAS** | **TRaC** | **TAS** | **TRaC** | **TAS** |
| Accessibility to cities | 0.32 (-0.01, 0.65) | **0.36 (0.04, 0.67)** | **0.19 (0.01, 0.38)** | **0.21 (0.06, 0.35)** | 0.13 (-0.07, 0.32) | 0.22 (0.00, 0.43) |
| Air temperature | -0.33 (-0.75, 0.10) | -0.12 (-0.63, 0.41) | -0.17 (-0.40, 0.07) | -0.03 (-0.24, 0.18) | -0.03 (-0.27, 0.20) | -0.14 (-0.43, 0.14) |
| Distance to water* | -0.06 (-0.30, 0.17) | 0.15 (-0.14, 0.42) | **0.07 (0.07, 0.21)** | 0.04 (-0.09, 0.16) | 0.10 (-0.04, 0.24) | -0.07 (-0.25, 0.10) |
| Elevation | **-0.50 (-0.94, -0.08)** | -0.38 (-0.95, 0.17) | **-0.39 (-0.62, -0.16)** | **-0.35 (-0.58, -0.13)** | **-0.36 (-0.60, -0.13)** | **-1.10 (-1.43, -0.77)** |
| NDVI | -0.20 (-0.55, 0.14) | 0.21 (-0.07, 0.50) | 0.02 (-0.16, 0.20) | -0.03 (-0.14, 0.08) | -0.01 (-0.18, 0.16) | -0.02 (-0.14, 0.12) |
| Population density | -0.06 (-0.50, 0.35) | 0.13 (-0.13, 0.37) | 0.01 (-0.23, 0.25) | -0.08 (-0.21, 0.05) | 0.01 (-0.22, 0.23) | -0.05 (-0.19, 0.09) |
| Rainfall | 0.29 (-0.01, 0.59) | 0.06 (-0.30, 0.41) | 0.11 (-0.06, 0.28) | -0.01 (-0.16, 0.14) | 0.04 (-0.13, 0.21) | 0.14 (-0.06, 0.35) |

*Nearest mapped river, lake, or stream

NDVI: Normalized difference vegetation index

Bold numbers indicate statistical significance at an alpha level of 0.05. All covariates have been centered and standardized.
