# Supplementary material for: Geospatial analysis of Plasmodium falciparum serological indicators: school versus community sampling in a low-transmission malaria setting
Source: BMC Med. 2024 Jan 23;22:31. doi: 10.1186/s12916-023-03145-6 (PMC10804471; doi:10.1186/s12916-023-03145-6)
Supplement: Supplementary file 2 — Additional file 2: Fig S1. Triangulated meshes used to build the stochastic partial difference equation (SPDE) models. Figure S2. Number of participants with a positive rapid diagnostic test (RDT) for malaria by study site. Figure S3. IgG seropositivity by age for Tracking Results Continuously (TRaC) community surveys in Haiti, 2012-2017. Figure S4. Correlation in the observed IgG levels among the LSA-1, MSP1, and AMA1 antigens utilized in this study for both survey types. Figure S5. Correlations between observed and predicted seroprevalence to (A) LSA-1, (B) AMA1, and (C) MSP1 based on model validation. Figure S6. Predicted seroprevalence across Haiti to (A) LSA-1, (B) AMA1, and (C) MSP1, aggregated for each commune. [file 12916_2023_3145_MOESM2_ESM.docx]

**Additional File 2**

**
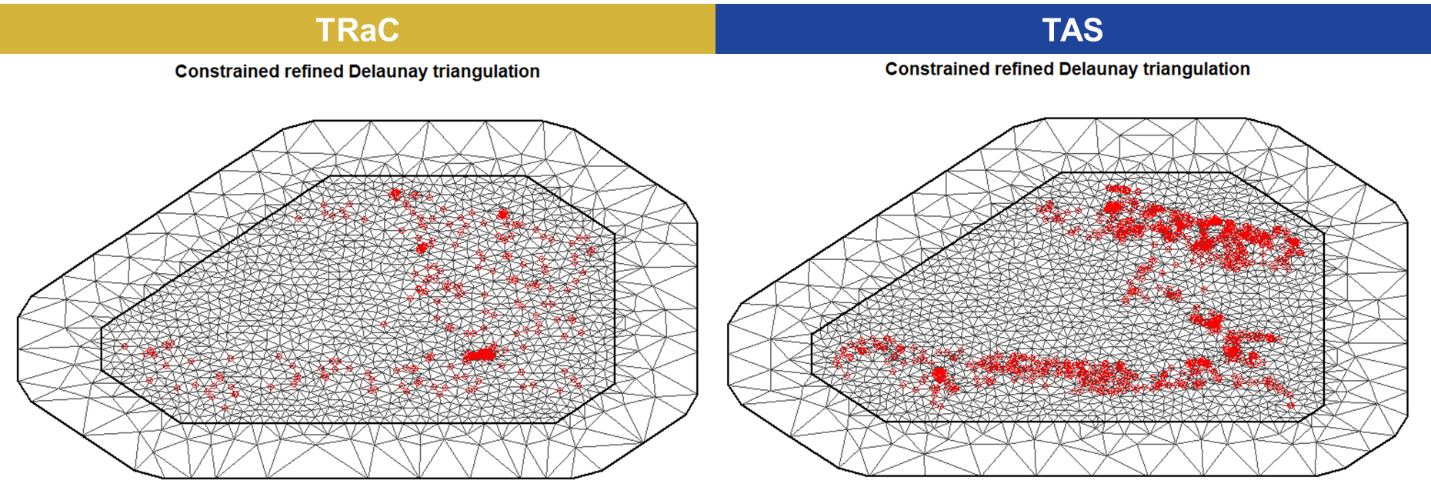
**

**Figure S1. Triangulated meshes used to build the stochastic partial difference equation (SPDE) models.** Red dots represent study sites.

**
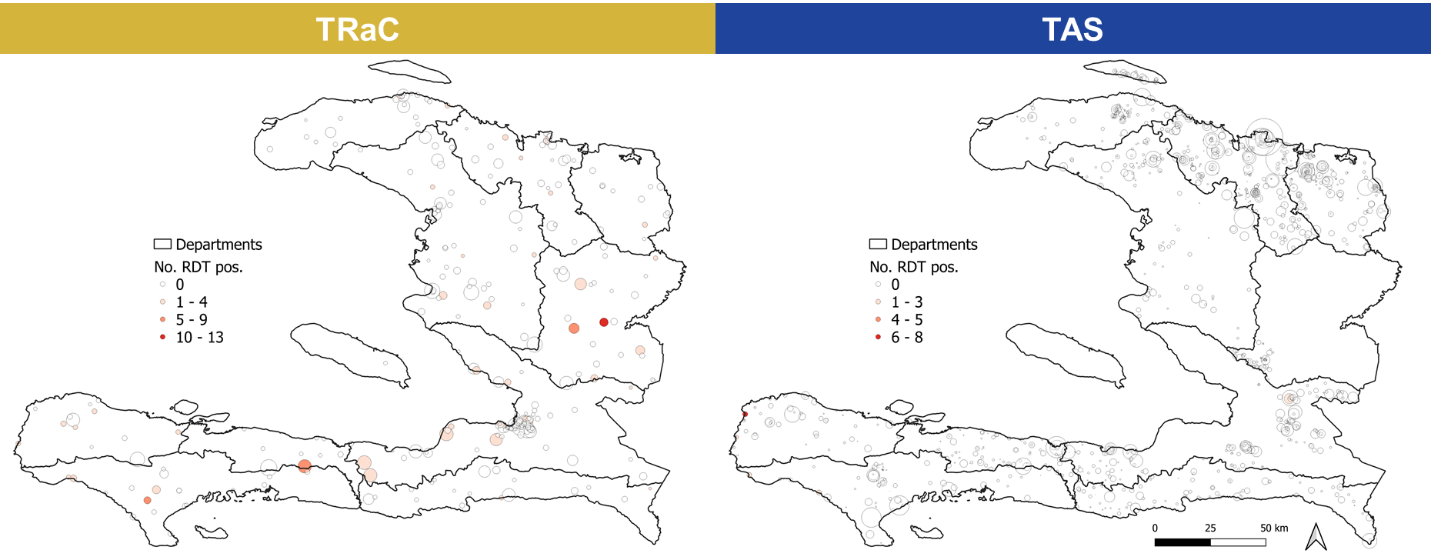
**

**Figure S2. Number of participants with a positive rapid diagnostic test (RDT) for malaria by study site.** Tracking Results Continuously (TRaC) sites are depicted on the left, and Transmission Assessment Survey (TAS) sites on the right. Circle sizes are proportional to the number of participants surveyed at each site. Darker red shading indicates higher numbers of positive RDTs; white fill indicates zero participants were positive at a given site. Department boundaries are outlined.


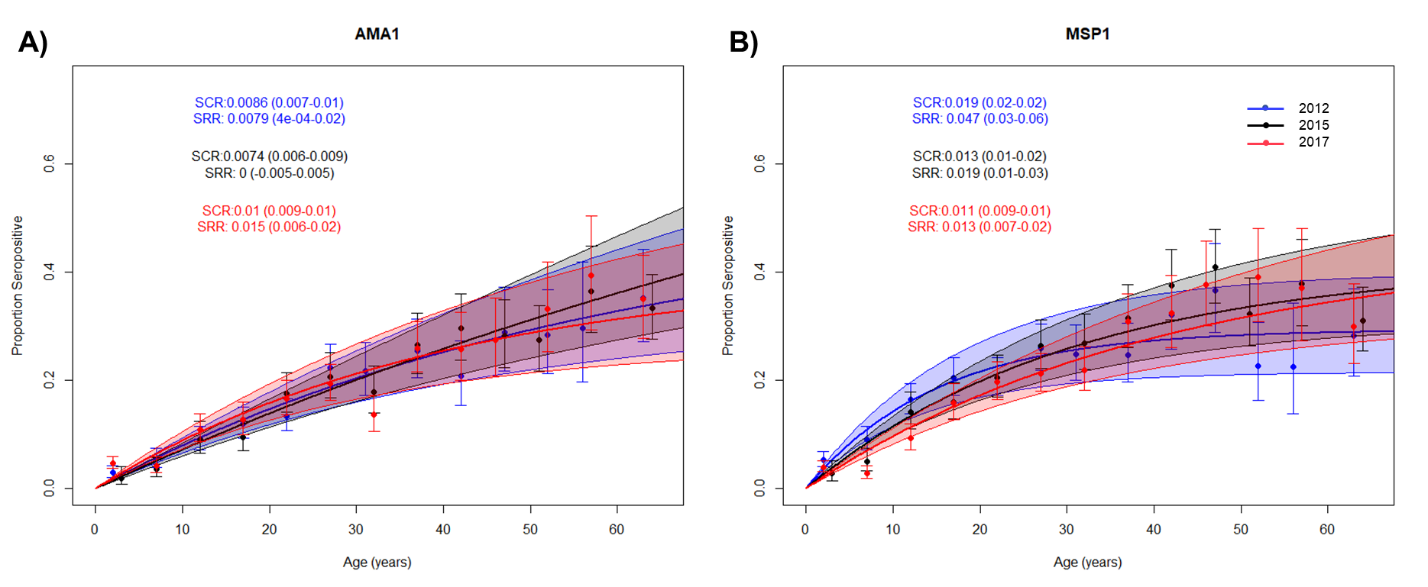


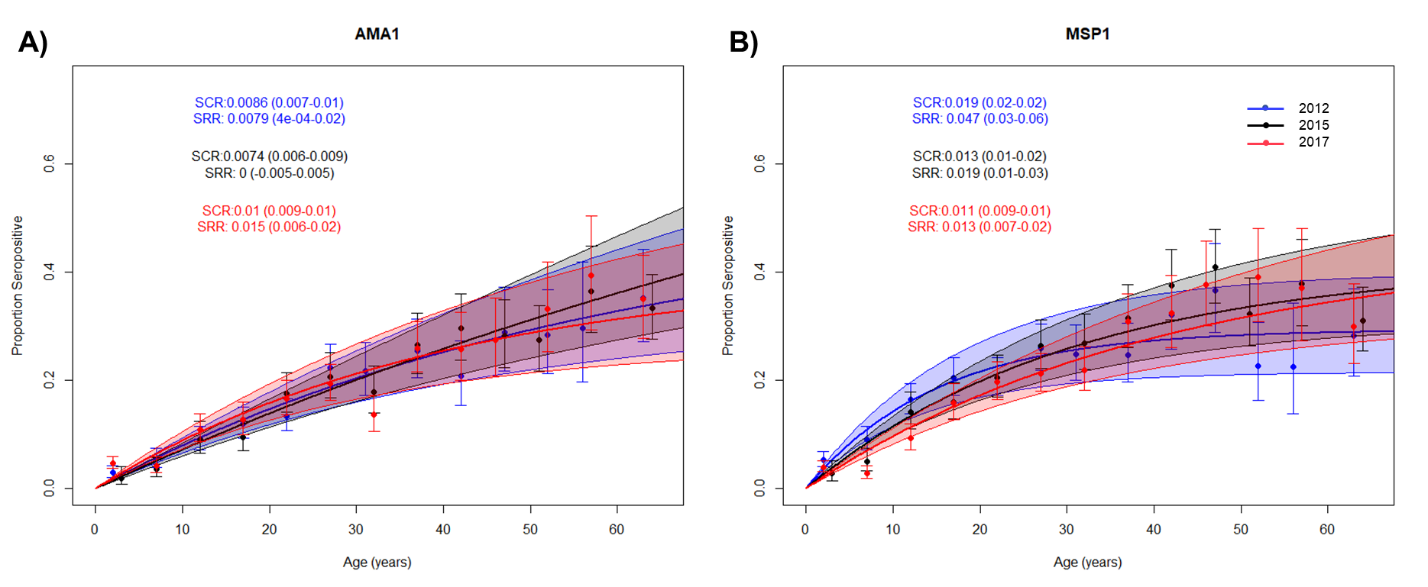


**Figure S3. IgG seropositivity by age for Tracking Results Continuously (TRaC) community surveys in Haiti, 2012-2017.** Points represent seropositivity estimates and 95% confidence intervals for seropositivity within each age category. Curves represent the fit of a catalytic conversion model, and shading indicates 95% confidence intervals of the model fit. Serological conversion rate (SCR) and serological reversion rate (SRR) estimates are provided for each year: blue, 2012; black, 2015; red, 2017.


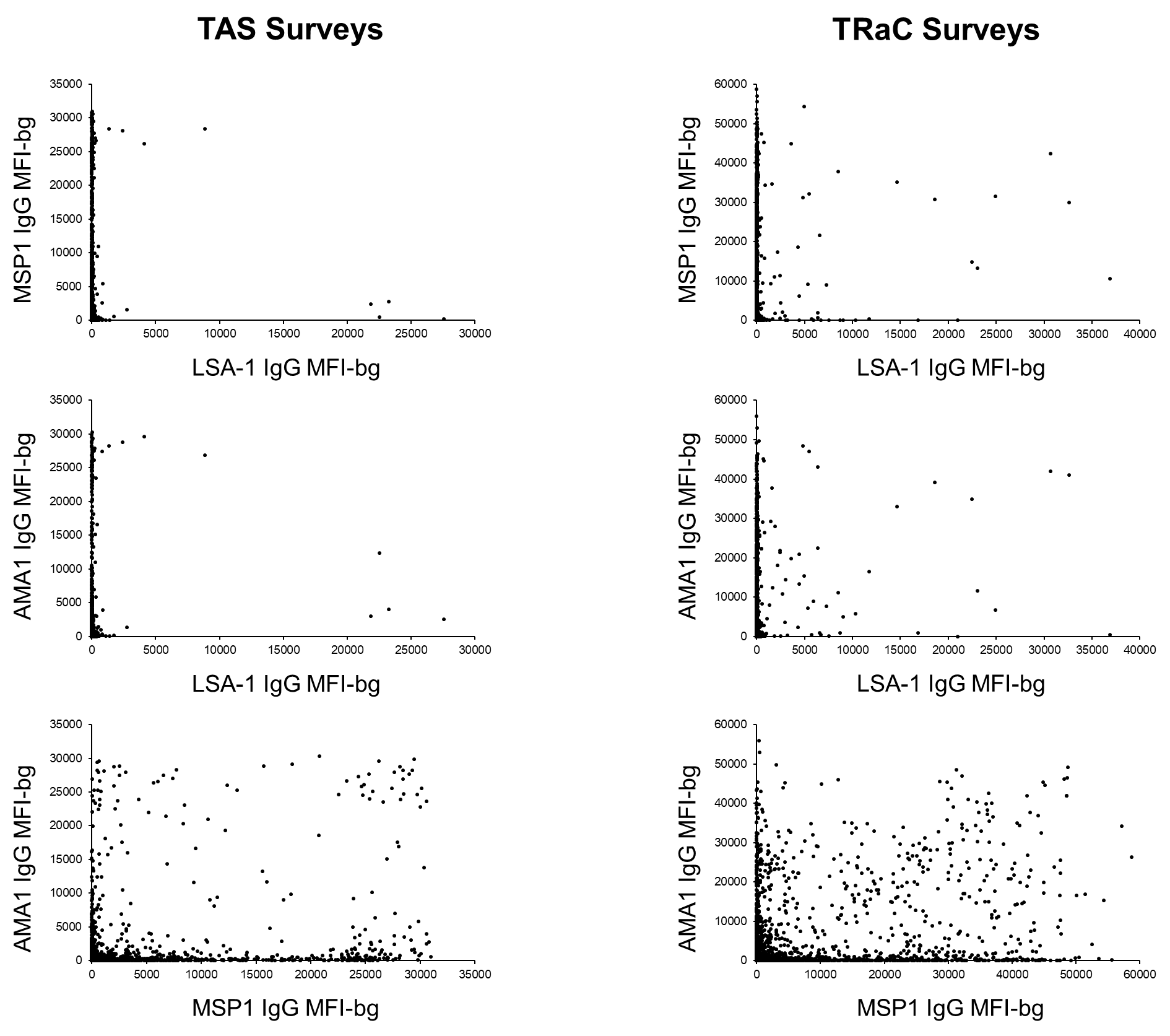


**Figure S4. Correlation in the observed IgG levels among the LSA-1, MSP1, and AMA1 antigens utilized in this study for both survey types.** Left panels show the median fluorescence intensity minus background (MFI-bg) IgG level among all three combinations of antigens for the TAS survey participants, while the right panels show the correlation for the TRaC survey participants.


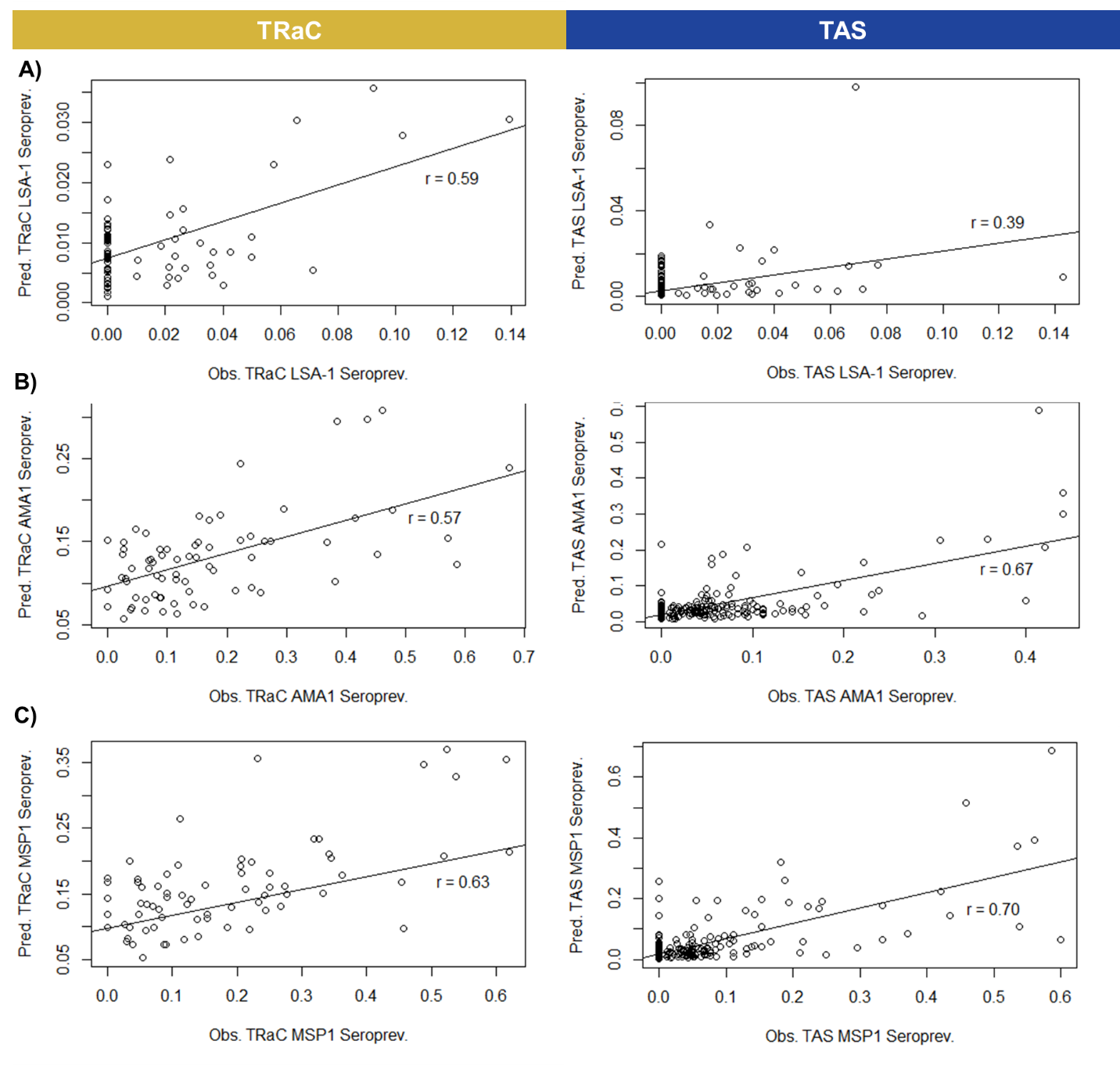


**Figure S5.** **Correlations between observed and predicted seroprevalence to (A) LSA-1, (B) AMA1, and (C) MSP1 based on model validation.** Tracking Results Continuously (TRaC) plots are depicted on the left and Transmission Assessment Survey (TAS) on the right. Each plot includes a linear regression line and Pearson’s correlation coefficient, *r*, to indicate the strength and direction of the relationship between observed and predicted seroprevalence values from validation datasets. These datasets were generating using a cross-validation approach by splitting TRaC and TAS data into 75% training and 25% testing, such that approximately 224 TRaC survey site data points and 845 TAS data points were used to predict the remaining 74 and 282 data points, respectively. These predictions were then compared to what was actually observed for the 25% testing set, yielding correlation coefficients for assessment of each model’s predictive performance. A correlation coefficient of +1 would indicate that each predicted data point perfectly correlates with each corresponding observed point.


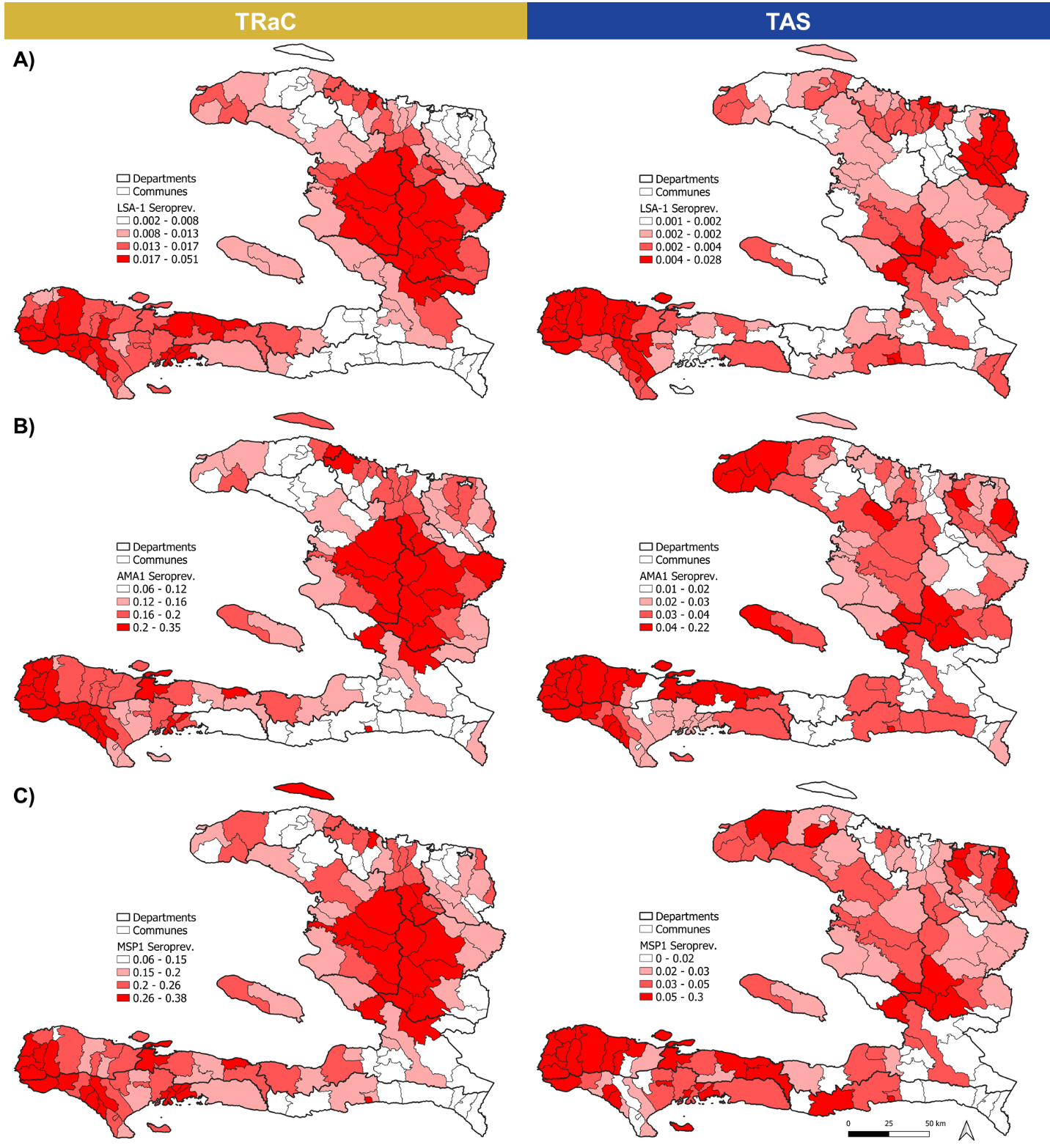


**Figure S6.** **Predicted seroprevalence across Haiti to (A) LSA-1, (B) AMA1, and (C) MSP1, aggregated for each commune.** Tracking Results Continuously (TRaC) predictions are on the left-hand side and Transmission Assessment Survey (TAS) on the right. Darker red shading indicates higher seroprevalence. Department boundaries are outlined in thick lines and commune boundaries in thin lines.
